# Text-Book of Anatomy

**Published:** 1907-06

**Authors:** 


### Reviews of Books.

**Text-Book of Anatomy.** Edited by D. J. CUNNINGHAM, M.D. Second Edition. Pp. xxxiii., 1388. Edinburgh: Young J. Pentland. 1906.

This a formidable work, larger much than its apparent bulk indicates, and perhaps too large for the average student.

As far as possible it has been written by various anatomists trained in the Edinburgh School, so that it may well be termed the Edinburgh Text-Book of Anatomy.

In this second edition many improvements have been made, especially in the illustrations, which may now rank with the finest in any text-book.

Additions and corrections have been made in nearly every section. In the section on "Osteology," written by Professor Arthur Thomson, one notices many additions, and we would like to suggest many more, as well as corrections. We have referred in the review of another work to the unsatisfactory account of the chondro-cranium, and all that has been said there may be said with greater emphasis here, for by now the works of Froriep, Jacobi, Levi, and others have established beyond all question the real facts of the development of the chondro-cranium, and shown that it very materially differs from the account originally given by Wiedersheim. The account, too, of the chorda dorsalis must undergo revision, more especially with regard to its relation to the basilar plate. The account of the ossification of the skull bones is very largely based on the observations of Rambaus and Renault. Many of these have not stood the test of time, and, as a consequence, accounts based on them will require revision. The systematic description of the bones is, on the whole, very satisfactory, although we think much more might have been made of the distinguishing features of certain of the vertebræ and ribs, with a view to enabling the student to identify them.

The section on joints is satisfactory, if not particularly interesting; there is little fault to be found with that on the muscular system, by Paterson; but we certainly question the accuracy of the markings of muscular attachments to the coronoid process, as figured on page 243, and the insertion of the supinator brevis is represented as a continuous area on the radius, which is well known not to be the case. However, these are small points. As to the section on the "Peripheral Nervous System," it may at once be declared to be one of the best in the book, not only from its accuracy, but from its interest.

As might be expected, the "Central Nervous System" receives authoritative treatment at the hands of the Editor, who has been careful to profusely illustrate it with a series of drawings of transverse sections of the brain stem of surpassing excellence. We know of none which equal them. Great care has been taken to keep the matter up to date, and we are glad to see due prominence given to the valuable work of Elliot Smith, as well as that of Flechsig and Bruce. The student has certainly enough food for reflection in this section.

The articles on the "Organs of Special Sense" are exhaustive, and have, perhaps, a savour of Pestut about them. We regret to see Jacobson's cartilages illustrated by a drawing of a section of the nose of a kitten, for the conditions in the kitten and man are vastly different. It has long formed a subject of discussion, this peculiar position of the Jacobsonian organ in man. Both in the textual description of the Jacobsonian organ, and in the chapter on "Embryology," this body is described as having a close relation to the Jacobsonian cartilage, whereas no such close relation exists, as may be seen in any coronal section of the human nose up to the third month.

It would be difficult to speak too highly of the chapter on "Embryology." It is admirably written and illustrated. Here and there a point or two might have been clearer, and the erroneous statements with reference to the Jacobsonian organ have already been alluded to. Fig. II. on page 41 is not credited to any animal, but certainly does not belong to man.

Birmingham's account of the digestive system remains much as he left it, and is an extremely accurate and interesting exposition of the "formalin" body.

The article on the "Lymphatics" has undergone a thorough revision, and has benefited materially by the publication of the great French work, *Poirier et Charpy.*

The book is completed by a section on "Surgical Anatomy," written by Stiles. This is a very satisfactory article. It brings into prominence Chiene's method of locating the various fissures and convolutions of the outer aspect of the hemisphere.

There will be found in the description of the prostate a curious contradiction of the description given by Dixon of the "capsule" of the prostate. Contradiction of this kind are almost inevitable in a book of this nature and magnitude, but this, we believe, is the only one in the book, which says much for the careful editing it has undergone.

It is not an easy task to "place" this book. Honestly, one hesitates to recommend it to the ordinary student; to the University student, or the student working for the "Fellowship" examination it ought to be essential, and as a guide to the researcher it is sufficiently stimulating. Its value to the last-mentioned would be greatly enhanced by a bibliography appended to each chapter.

